# Murine matrix metalloproteinase-20 overexpression stimulates cell invasion into the enamel layer via enhanced Wnt signaling

**DOI:** 10.1038/srep29492

**Published:** 2016-07-11

**Authors:** Masashi Shin, Maiko Suzuki, Xiaomu Guan, Charles E. Smith, John D. Bartlett

**Affiliations:** 1 Division of Biosciences, Ohio State University, College of Dentistry, Columbus, Ohio 43210, USA; 2 Dept. of Mineralized Tissue Biology and Harvard School of Dental Medicine, The Forsyth Institute, Cambridge, MA, 02142, USA; 3 Department of Anatomy & Cell Biology, Facility for Electron Microscopy Research, McGill University, Montreal, QC, H3A 0C7, Canada

## Abstract

Matrix metalloproteinase-20 (MMP20) is expressed by ameloblasts in developing teeth and *MMP20* mutations cause enamel malformation. We established a stably transfected *Tet*-Off *Mmp20*-inducible ameloblast-lineage cell line and found that MMP20 expression promoted cell invasion. Previously, we engineered transgenic mice (Tg) that drive *Mmp20* expression and showed that *Mmp20*^+/+^Tg mice had soft enamel. Here we asked if *Mmp20* overexpression disrupts ameloblast function. Incisors from *Mmp20*^+/+^ mice expressing the *Mmp20* Tg had a striking cell infiltrate which nearly replaced the entire enamel layer. A thin layer of enamel-like material remained over the dentin and at the outer tooth surface, but between these regions were invading fibroblasts and epithelial cells that surrounded ectopic bone-like calcifications. *Mmp20*^+/+^Tg mice had decreased enamel organ cadherin levels compared to the *Mmp20* ablated and WT mice and, instead of predominantly locating adjacent to the ameloblast cell membrane, β-catenin was predominantly present within the nuclei of invading cells. Our data suggest that increased cadherin cleavage by transgenic MMP20 in the WT background releases excess β-catenin, which translocates to ameloblast nuclei to promote cell migration/invasion. Therefore, we conclude that MMP20 plays a role in normal ameloblast migration through tightly controlled Wnt signaling and that MMP20 overexpression disrupts this process.

Matrix metalloproteinases (MMP) regulate cell movement, wound healing, tissue repair, regeneration, remodeling, morphogenesis and development^1^. MMP20 is expressed in teeth^2^,^3^,^4^,^5^ and the only non-overlapping function of MMP20 is in enamel formation^6^. Ablation of *Mmp20* in mice causes enamel to become thin, brittle and to flake off the underlying dentin^7^. In humans, seven different *MMP20* mutations are currently known to cause enamel malformation termed *amelogenesis imperfecta*^8^.

During tooth development, ameloblasts are responsible for enamel formation and odontoblasts are responsible for dentin formation. Ameloblasts are epithelial cells and odontoblasts are mesenchymal cells^9^,^10^ derived from the neural crest, which also have an epithelial origin^11^. The three major stages of enamel development are the secretory, transition and maturation stages. During the secretory stage, ameloblasts secrete enamel matrix proteins as thin crystallites are induced to grow out from the dentin surface. During the transition stage, the crystallites are at their full length and the ameloblasts change into shorter maturation stage cells that typically modulate between ruffle- and smooth-ended morphologies and actively reabsorb the extracellular enamel matrix proteins and their fragments. During the late maturation stage, virtually all protein is removed from the enamel layer and the crystallites have grown substantially in width and thickness^8^.

MMP20 is predominantly expressed by secretory stage ameloblasts and its expression decreases abruptly when the ameloblasts enter the maturation stage^2^,^3^,^4^,^5^. In rodent incisors, a papillary layer exists between maturation stage ameloblasts and the labial connective tissue^12^,^13^,^14^. The epithelial cells forming the papillary layer are separated from closely associated capillaries and fibroblasts by a basement membrane^13^, and these cells are thought necessary in part to help neutralize or remove acid stress generated as the enamel crystallites expand in volumetric size during maturation^15^.

Ameloblast cell-cell attachment, detachment and cell movement are regulated so that the characteristic rodent decussating enamel rod pattern can form during the secretory stage of amelogenesis^16^. Cadherins are stabilized by p120-catenin (p120) at the cell membrane so that cadherin extracellular domains can bind to one another to form stable cell-cell junctions^17^,^18^. In mice with p120 conditionally ablated from epithelial tissues, the ameloblasts lose polarity and detach from each other and their surrounding tissues resulting in severely malformed enamel^19^. Conditional ablation of E-cadherin from the incisor cervical loop region where stem cells reside, increases stem cell proliferation and decreases stem cell migration^20^. These studies highlight the importance of ameloblast cell-cell attachments during early enamel development. Previously, we demonstrated that *Mmp20* overexpression in mice results in significantly softer than normal dental enamel^21^. Here we ask if *Mmp20* overexpression leads to disruption of ameloblast function during enamel formation.

## Results

### Establishment of MMP20-overexpressing cell lines

**Figure 1:**
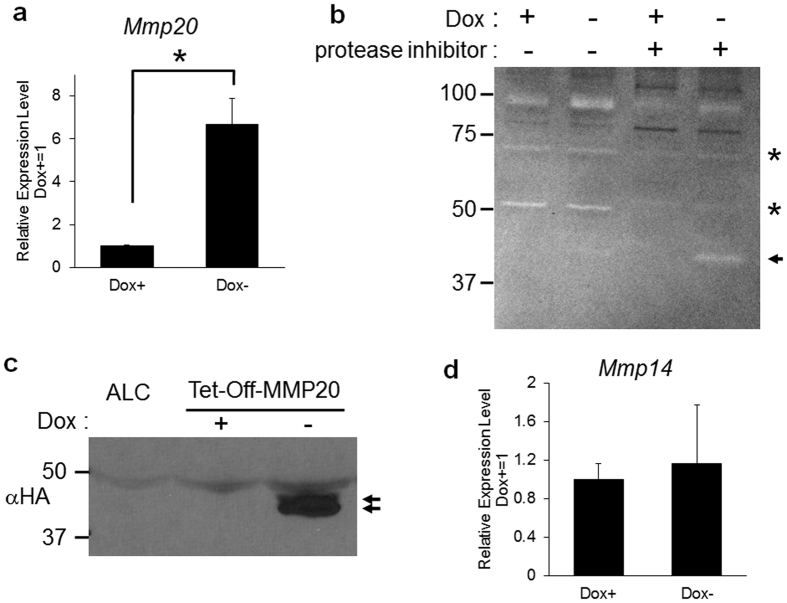
An inducible MMP20-overexpressing ameloblast-lineage (ALC) cell line. The murine *Mmp20* cDNA containing a Val^101^-Gly^101^ mutation for MMP20 auto-activation and encoding a downstream hemaggultinin tag (HA) for enhanced immunodetection, was ligated into the Tet-Off Advanced Inducible gene expression vector. ALC cells were stably transfected with this vector (*Tet*-Off-MMP20 cells) and were assessed for levels of MMP20 expression. (**a**) *Tet*-Off-MMP20 cells were incubated with or without 100 ng/ml Doxcycline (Dox) for 48 hours followed by collection of total RNA for qPCR expression analysis. *Mmp20* expression was approximately six fold higher in the Dox− as compared to the Dox+ medium (*P < 0.05). (**b**,**c**) *Tet*-Off-MMP20 cells were cultured in serum free medium with protease inhibitors, but not MMP inhibitors, to prevent MMP20 degradation. (**b**) Zymography demonstrated that in the absence of Dox, active MMP20 (arrow) was secreted into the culture medium and that protease inhibitors were necessary to visualize MMP20 activity. Asterisks denote inhibited proteases. (**c**) Immunoblot of the MMP20 HA tag demonstrating high level MMP20 expression (arrows) in the absence of Dox. (**d**) Negative control qPCR demonstrating no significant difference in *Mmp14* expression levels in the presence or absence of Dox.

Although there are a few published reports to the contrary, no established tooth cell line exists that expresses appreciable amounts of MMP20. We therefore engineered ameloblast lineage cells (ALC)^22^ to inducibly express active MMP20 in the Tet-Off system. *Mmp20* expression levels in the generated cell lines were quantified by qPCR. *Mmp20* was expressed approximately 6 fold higher in the absence of doxycycline (Fig. 1a). Initially, we were unable to detect MMP20 in the culture medium of induced cells. However when we added a protease inhibitor cocktail (without MMP inhibitors) to the culture medium, MMP20 was then detectable by zymography (Fig. 1b) and by immunoblotting with an antisera specific for the engineered HA tag at the C-terminus of the MMP20 protein (Fig. 1c). qPCR analysis of membrane-type-1 MMP (MT1-MMP, *Mmp14*) expression served as the negative control for *Mmp20* inducible expression (Fig. 1d).

### MMP20 increases ALC cell invasion

**Figure 2:**
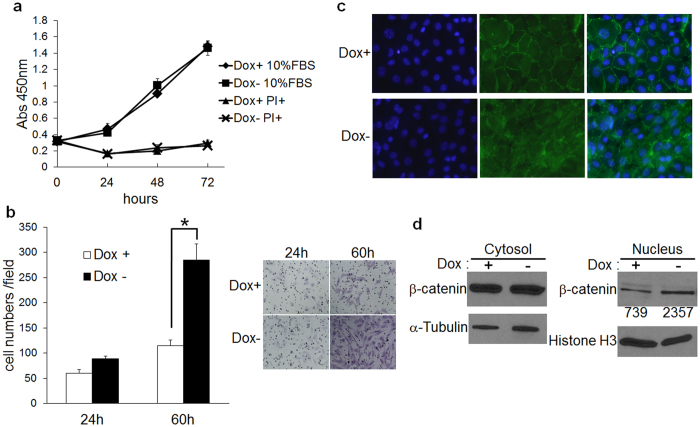
Analysis of cell proliferation and invasion with and without MMP20 expression. (**a**) Cell proliferation was quantified by the WST-1 assay that measures dye formation by mitochondrial enzymes. No difference in cell proliferation was observed as a function of MMP20 expression over time in the presence or absence of fetal bovine serum (FBS). PI, protease inhibitors excluding MMP inhibitors. (**b**) *Tet*-Off-MMP20 cells were added to filter well inserts (8 μm pore size) coated with Matrigel and were placed into culture wells. After incubation for 24 h or 60 h in serum free medium with non-MMP protease inhibitors, the filters were washed, fixed and hematoxylin stained. Five random microscope cell fields per insert were counted under a microscope and the cell counts showed that more cells migrated to the opposite side of the filter when MMP20 was expressed (Dox−) compared to when MMP20 was not expressed (Dox+). Data are the mean ± SD for three independent experiments. *P < 0.05. The panel on the right shows example cell fields for each time and treatment. (**c**) *Tet*-Off-MMP20 cells were seeded on glass coverslips with or without 100 ng/ml Dox for 48 h. Immunofluoresence identified β-catenin location and DAPI staining identified cell nuclei. β-catenin localization adjacent to cell membranes was disrupted with MMP20 expression (Dox−). (**d**) Immunoblots were performed on β-catenin extracted from the cell cytosol or nucleus after 24 h treatment with or without Dox. More β-catenin was present within the cell nuclei when MMP20 was expressed (Dox−). Loading control proteins were α-tubulin for the cytosol and histone H3 for the nucleus. Numbers indicate relative band intensities after scanning.

Ameloblasts move in groups that slide by one another as the enamel layer thickens (secretory stage of development), and this movement culminates in the characteristic decussating enamel prism pattern observed in rodent incisors^23^ or the entwined gnarled prism pattern seen in human teeth^24^. To determine if MMP20 promotes cell invasion *in vitro*, we performed a trans-well invasion assay where Tet-off-*Mmp20* ALC cells migrate through a Matrigel coated membrane with and without *Mmp20* induction. For invasion studies, all cells were treated with broad spectrum protease inhibitors containing no MMP inhibitors. Although no difference in cell proliferation existed between induced and uninduced cells (Fig. 2a), a significant difference in cell invasion was observed (p < 0.05). More cell migration through the Matrigel membrane was observed in ALC cells induced to express MMP20 versus the uninduced cells (Fig. 2b).

It was demonstrated previously that when MMPs cleave an extracellular classic cadherin domain, β-catenin is removed from its position near the cell membrane and in many cases translocates to the cell nucleus^25^,^26^,^27^,^28^,^29^,^30^,^31^. This translocation is associated with cell movement^32^,^33^,^34^. MMP20 cleaves cadherins^35^ so we asked if *Mmp20* expression removes β-catenin from the Tet-off-*Mmp20* ALC cell membrane. Immunocytochemistry results demonstrated a striking disruption of membrane bound β-catenin in ALC cells induced to express *Mmp20* (Fig. 2c). Immunoblots showed that more β-catenin was present in cell nuclei when *Mmp20* was induced (Fig. 2d). These results indicate that WNT signaling likely plays a role in the observed increase in cell invasion.

### Tissue specificity of *Mmp20* transgene expression

The *Mmp20* transgene has 4.6 kb of mouse amelogenin promoter inserted 5′ to the mouse *Mmp20* cDNA and has 1.1 kb of amelogenin non-coding region that includes amelogenin polyadenylation sites inserted immediately downstream of the *Mmp20* cDNA^21^. The transgenic mice used for all experiments reported here had the most highly expressing incisor transgene (Tg24)^21^. *Mmp20* is normally expressed only in the enamel organ and pulp of teeth^36^. We therefore asked if this transgene maintained its tissue restricted pattern of expression. *Mmp20* quantitative real-time PCR (qPCR) of tissues from transgene positive mice in the wild-type background (*Mmp20*^+/+^Tg) revealed that of nine tissues assessed, only mouse incisors expressed appreciable amounts of *Mmp20* mRNA (Supplementary Table S1).

### Developmental pattern of *Mmp20* transgene expression

**Figure 3:**
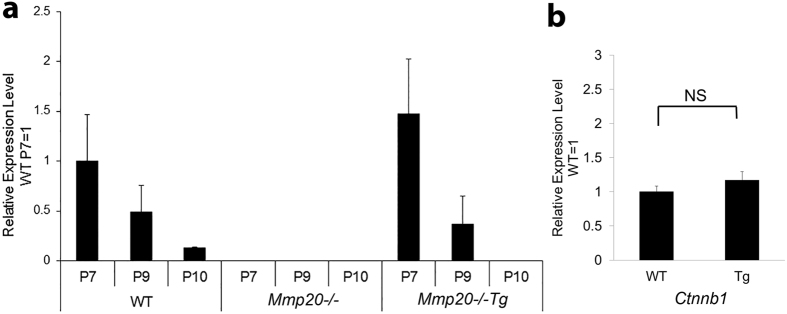
Relative expression levels of *Mmp20* in wild-type (WT) and *Mmp20*^−/−^Tg enamel organ. Total RNA was isolated from first molar enamel organs and pulp of 7-day-old (P7, late secretory-early maturation stage), 9-day-old (P9, early-mid maturation stage) and 10-day-old (P10, mid-maturation stage) pups. (**a**) The mouse genotypes examined were WT, *Mmp20*^−/−^ and *Mmp20*^−/−^Tg. *Mmp20* expression was quantified by qPCR and as expected, no expression was observed in the *Mmp20*^−/−^ mice. In WT mice, *Mmp20* was expressed in the enamel organ and expression declined with each successive developmental stage. The amelogenin promoter driven transgene in the *Mmp20*^−/−^Tg had an *Mmp20* expression pattern similar to that of WT. (**b**) β-catenin (*Ctnnb1*) expression levels were not significantly (NS) increased in enamel organs from *Mmp20*^+/+^Tg (Tg) mice compared to WT.

*Mmp20*^−/−^Tg first molars were collected at postnatal day 7 (late secretory-early maturation stage), day 9 (early-mid maturation stage) and day 10 (mid maturation stage). Enamel organs from wild-type (WT) mice showed a decreasing *Mmp20* expression level in enamel organ as development progressed. In contrast, enamel organs from *Mmp20*^−/−^ mice did not express *Mmp20*. *Mmp20*^−/−^Tg mice had higher than WT enamel organ *Mmp20* expression that, like WT mice, diminished with progressing developmental stages (Fig. 3a). So, with the exception of higher than normal expression levels, the transgene was developmentally regulated in a similar manner to WT *Mmp20* expression. We found that β-catenin (*Ctnnb1*) expression levels in enamel organs from *Mmp20*^+/+^*Tg mice* were not increased over WT levels (Fig. 3b). So, if β-catenin promotes cell migration/invasion *in vivo*, the β-catenin would originate from cleavage and disruption by MMP20 of the cadherin/catenin complex.

### Morphological analyses of *Mmp20* ^+/+^Tg enamel

**Figure 4:**
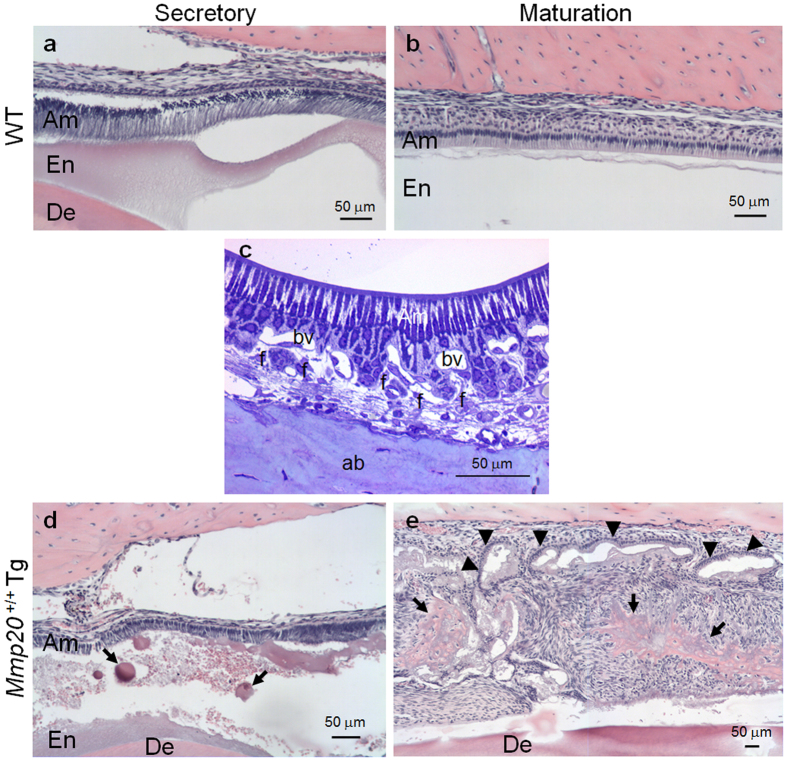
Identification of a striking cell infiltrate present within the *Mmp20*^+/+^Tg mouse incisor enamel space. Demineralized longitudinal incisor sections from (**a**) wild-type (WT) secretory and (**b**) WT maturation stages. (**c**) Semithin sections of plastic embedded samples consisting of maturation stage enamel organs from WT mice were stained with toluidine blue. The outer surface of the papillary layer of the enamel organ is intimately associated with blood vessels (bv) and other connective tissue elements including fibroblasts (f) extending out to the labial alveolar bone (ab). (**d**) In contrast to WT, the *Mmp20*^+/+^Tg secretory stage incisors had ectopic calcifications in the enamel space (arrows). (**e**) The *Mmp20*^+/+^Tg maturation stage incisors had a profound pathology consisting of a massive cell infiltrate, extensive ectopic calcifications (arrows) and a disorganized ameloblast layer that appeared to weave in and out of the sectioning plain (arrow heads).

Incisors from WT and *Mmp20*^+/+^Tg mice were demineralized, longitudinally sectioned and stained with H&E (Fig. 4a,b,d,e). In WT mice, secretory stage enamel organ had tall columnar ameloblasts and enamel matrix proteins were present in the forming enamel (Fig. 4a). During the maturation stage, WT mice had the characteristic shortened ameloblasts and virtually all the protein was removed from the enamel space (Fig. 4b). In contrast, the *Mmp20*^+/+^Tg secretory stage enamel had a rough/spotty protein content with ectopic calcifications (Fig. 4d). Surprisingly, the *Mmp20*^+/+^Tg maturation stage enamel organ had highly irregular cyst-like rows of ameloblasts that appeared to weave in and out of the section plain (Fig. 4e, arrowheads), and had a massive cell infiltrate present within what should have been an enamel space. Ectopic bone-like calcifications (Fig. 4e, arrows) with embedded cells were also present within the cell infiltrate. The large spindle shaped cell infiltrate and large ectopic calcifications suggested that fibroblasts normally residing within the infoldings of the papillary layer^12^,^13^,^14^ (Fig. 4c) had mostly replaced the typical enamel layer. Trichrome staining of WT and *Mmp20*^+/+^Tg enamel (Supplementary Fig. S1a,b) revealed the presence of a foamy mineral present within the *Mmp20*^+/+^Tg enamel space. Next, we performed immunohistochemistry to determine if type I collagen was present. Type I collagen was identified in dentin from both WT and *Mmp20*^+/+^Tg mice (Supplementary Fig. S1c,d). Strong expression was identified along the dental-enamel junction, which may have been an edge effect. However, Type I collagen was also present within the enamel space of just the *Mmp20*^+/+^Tg mice where the invading cells accumulated, suggesting that the ectopic calcifications do contain collagen. These are striking results given that overexpression of an endogenous MMP caused such a severe pathology.

### An autoimmune response to amelogenin was not detectable and may not be responsible for the severe pathology present in the *Mmp20* ^+/+^Tg enamel organ

To rule out the possibility that the enamel organ pathology observed in *Mmp20*^+/+^Tg mice was caused by a strong autoimmune response to amelogenin, we performed ELISA assays to identify anti-amelogenin antibodies within serum from *Mmp20*^+/+^Tg mice (Supplementary Fig. S2a). The assay included negative control serum from WT mice, a no serum control with the secondary antibody added, a positive control containing anti-amelogenin antisera and a control identical to the positive control, but without the anti-amelogenin antisera. The controls worked as expected and no anti-amelogenin antibodies were detected in Tg mouse serum.

We next performed immunoblots on Tg mouse serum to confirm that amelogenin was not “leaking” into the bloodstream as a result of the pathology observed in the Tg mouse enamel organ. Proteins extracted from five-day-old mouse first molars served as the positive control for immunodetection of amelogenin. For each of the WT and Tg lanes, 0.1 and 0.2 μl of serum were loaded, electrophoresed and immunoblotted for the presence of amelogenin (Supplementary Fig. S2b). We did not detect amelogenin within the Tg serum suggesting that intact amelogenin was not present in high levels within Tg mouse blood.

### Identification of cell types within the infiltrate

**Figure 5:**
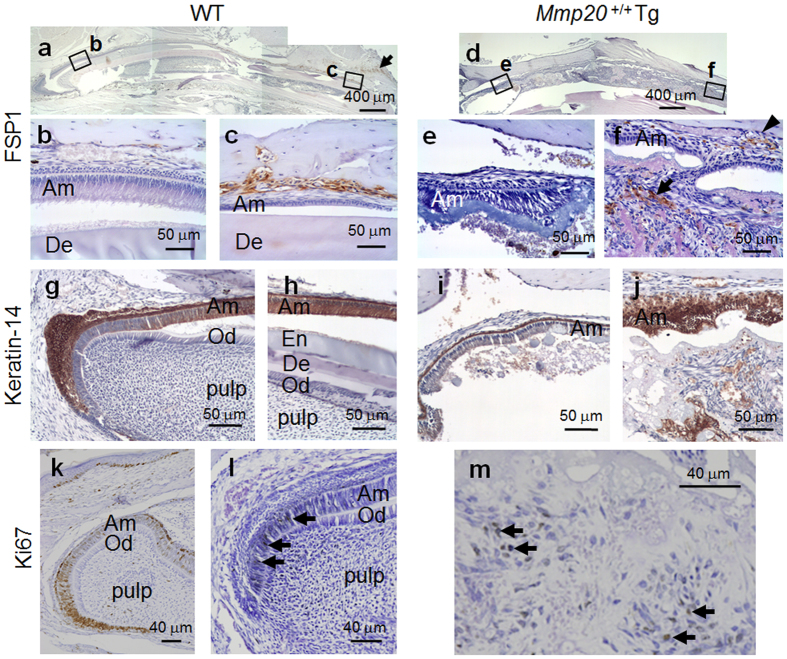
Localization of fibroblast specific protein-1 (FSP1) and Keratin-14 (K14) positive cells, and identification of proliferating cells via Ki67 expression in wild-type (WT) and *Mmp20*^+/+^Tg mouse incisors. Immunostaining for FSP1 was performed on longitudinal incisor sections. (**a**–**c**) Incisor sections from WT mice. FSP1 positive cells were detected in tissues near the (**a**, arrow) lips and in the loose connective tissues and (**b**,**c**) vacular spaces near labial bone. (**d**–**f**) Incisor sections from *Mmp20*^+/+^Tg mice. (**d**) The cell infiltrate in the Tg incisors became abundant soon after what appears to be the mid-late secretory stage (**e**,**f**). Within the cell infiltrate, FSP1 positive cells were present near the (**f**, arrow) ectopic bone-like mineral and near the (**f**, arrowhead) labial alveolar bone. Immunohistochemical staining of K14 was performed on longitudinal incisor sections from (**g**,**h**) WT and (**i**,**j**) *Mmp20*^+/+^Tg mice. (**g**) In WT mice, the enamel organ, but not the pulp stained positively for K14. Closer inspection revealed that pre-ameloblasts did not express K14. However, (**h**) maturation stage ameloblasts were K14 positive. (**i**,**j**) In *Mmp20*^+/+^Tg mice, K14 was present in the enamel organ, but was not strong until the equivalent, by tooth length, of what is the maturation stage of development in WT mice. (**i**) The secretory stage stratum intermedium stained strongly and the ameloblasts stained lightly. (**j**) However, during the maturation stage, the entire enamel organ was strongly positive for K14 and the cell infiltrate also had islands of strong staining. (**k**) In WT 3-day-old second molars and (**l**) WT adult incisors, Ki67 expression was observed in nuclei of pre-ameloblasts at the apical cervical loop (arrows). In contrast, (**m**) in *Mmp20*^+/+^Tg incisors, the cell infiltrate contained cells positive for nuclear Ki67 expression (arrows) indicating that a portion of the infiltrated cells were undergoing cell division. Ameloblasts, Am; odontoblasts, Od; enamel space, En and dentin, De.

To determine if the cell infiltrate contained fibroblasts, immunohistochemistry was performed with an antisera specific for fibroblast specific protein (FSP1)^37^. In incisors from WT mice (Fig. 5a–c), FSP1 was present within the intravascular areas of bone and within the regions of the labial connective tissue at the surface of the labial bone (Fig. 5c). Incisor enamel organs from *Mmp20*^+/+^Tg mice (Fig. 5d–f), showed little staining during the secretory stage (Fig. 5f), but strong FSP1 staining was observed during the tooth-length equivalent of the maturation stage in regions close to the labial bone (Fig. 5f, arrowhead) and within the infiltrate filling the enamel space, especially in areas around ectopic mineral deposits (Fig. 5f, arrow).

Since all the cells in the infiltrate did not stain positively for fibroblast specific antigen, we asked if epithelial cells were also present within the infiltrate. Keratin-14 (K14) is expressed by the epithelial-derived enamel organ^38^ and antisera specific for K14 was used to stain incisor sections from WT (Fig. 5g,h) and *Mmp20*^+/+^Tg mice (Fig. 5i,j). As was observed previously^39^, WT pre-ameloblasts did not express K14 although all other epithelial cell layers of the enamel organ were strongly positive (Fig. 5g). However, strong staining was observed in maturation stage ameloblasts (Fig. 5h). In incisor sections from *Mmp20*^+/+^Tg mice, the secretory stage enamel organ stained strongly except for ameloblasts (Fig. 5i), suggesting that they did not transition well from pre-secretory to secretory stage ameloblasts. However, the poorly organized maturation stage *Mmp20*^+/+^Tg ameloblasts did stain strongly for K14. Furthermore, the cell infiltrate also had strongly staining islands of K14-positive cells, some of which appeared near the ectopic calcifications (Fig. 5j).

Since the cell infiltrate was so massive, we asked if the cells within the infiltrate were undergoing cell division. The Ki67 protein is present during all active phases of the cell cycle G1, S, G2, and mitosis, but is absent from resting cells (G0), so it is an excellent marker for identifying the growth fraction of a given cell population^40^. We therefore used antisera specific for Ki67 to identify dividing cells within developing wild-type and MMP20 overexpressor teeth (Fig. 5k–m). The apical cervical loop region of the inner enamel epithelium contains stem cells that divide and differentiate into ameloblasts^20^. It was in this region of WT mouse molars (Fig. 5k) and incisors, that positive Ki67 staining was observed (Fig. 5l, arrows). The cell infiltrate present in the enamel space of *Mmp20*^+/+^Tg mice also had many cells undergoing cell division (Fig. 5m, arrows). So, the infiltrated cell mass was enlarged by cell growth within the mass.

### Gene expression in wild-type and *Mmp20* ^+/+^Tg mouse incisors

**Figure 6:**
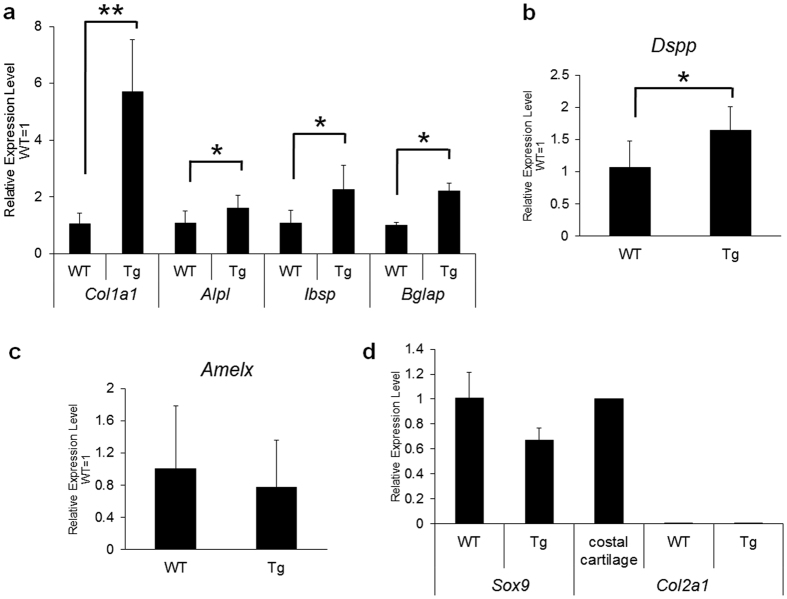
Expression levels of bone-related genes in wild-type (WT) and *Mmp20*^+/+^Tg (Tg) mouse incisor enamel. Whole incisors were ground, extracted for total RNA and prepared for qPCR quantification of gene expression. (**a**) Significantly increased transcript levels of alkaline phosphatase (*Alpl*), osteocalcin (*Bglap*), bone sialoprotein (*Ibsp*) and a highly significant increase in collagen 1α1 chain (*Col1a1*) transcripts were observed in incisors from *Mmp20*^+/+^Tg mice versus WT mice. (**b**–**d**) A significant increase in expression was observed for dentin sialophosphoprotein (*Dspp*), but not for amelogenin (*Amelx*), *Sox9* or collagen 2α1 (*Col2a1*) in the Tg mice relative to the WT controls. These expression differences were consistent with formation of bone-like calcifications observed within the enamel space of the Tg mice. *P < 0.05; **P < 0.01.

We asked if gene expression of bone-related proteins was induced within the transgenic incisors. Type I collagen (*Col1a1*) and integrin-binding sialoprotein (*Ibsp*) are secreted into extracellular matrices. Alkaline phosphatase (*Alpl*) induces mineralization of osteoid and gamma-carboxyglutamic acid protein (osteocalcin, *Bglap*) is a major non-collagenous protein synthesized by osteoblasts and cementoblasts^41^. To quantify expression levels of these genes, RNA was extracted from whole incisors for qPCR analysis. Compared to WT controls, increased gene expression was observed for *Col1a1* (P = 0.002), *Alpl* (P = 0.027), *Ibsp* (P = 0.03) and *Bglap* (P = 0.016) (Fig. 6a). Dentin sialophosphoprotein (*Dspp*) is expressed in odontoblasts and preameloblasts^5^,^42^,^43^ and was expressed at significantly higher levels in *Mmp20*^+/+^Tg incisors (P = 0.011) compared to WT incisors (Fig. 6b). However, amelogenin expression did not significantly differ between WT and *Mmp20*^+/+^Tg (Fig. 6c). We next asked if genes associated with condrogenesis were induced within transgenic incisors. *Sox9* expression did not significantly differ between genotypes and *Col2a1* expression was not detected in WT or *Mmp20*^+/+^Tg incisors (Fig. 6d). It is noteworthy that the biggest difference in gene expression between genotypes was for type I collagen as most evidence suggests that normal developing enamel does not contain type 1 collagen^44^. These results suggest that cell signaling pathways essential for enamel development are disrupted in the *Mmp20* overexpressing mice.

Since both *Col1a1* and *Dspp* expression are induced by *Wnt* signaling^45^,^46^, we asked if other *Wnt* responsive genes were also induced in *Mmp20*^+/+^Tg incisor enamel organs compared to incisor enamel organs from WT animals. By use of qPCR, we compared expression levels of *Myc, Lef1, Ccnd1* (Cyclin D1)*, Msx2, Dlx3, Fst* (follistatin), *Snail1, Slug,* and *Nog.* Surprisingly, we did not find significant differences in expression levels between WT and Tg mice for any of the genes examined (Supplementary Fig. S3). It is possible that subtle differences exist that we could not detect as the enamel organ is composed of several tissue layers and MMP20 is primarily secreted from just the single cell layer of ameloblasts.

### Former ameloblasts are part of the cell infiltrate present in the enamel space of the *Mmp20* overexpressing mice

**Figure 7:**
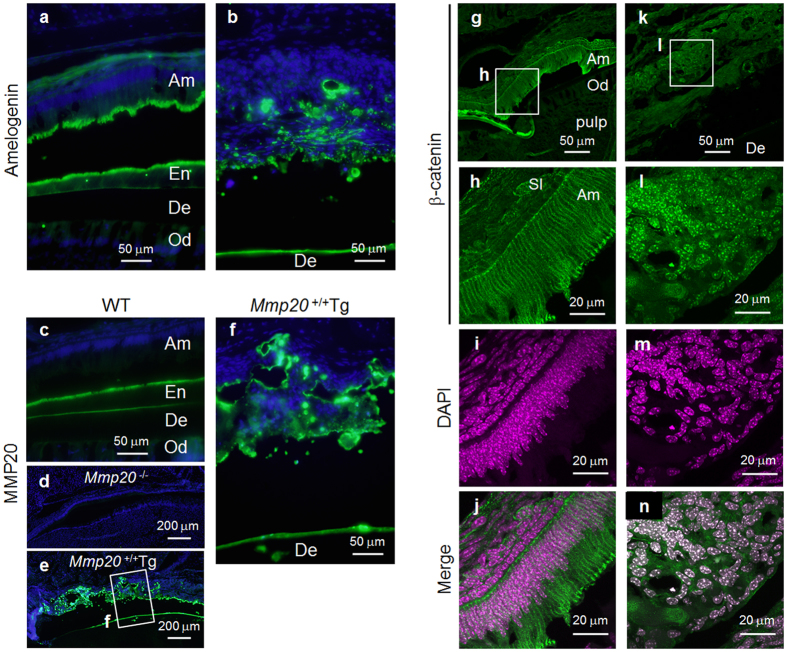
Amelogenin and MMP20 expression plus β-catenin localization in wild-type (WT) and *Mmp20*^+/+^Tg incisors. (**a**) Immnofluorescence localization confirmed that WT secretory stage ameloblasts express amelogenin and demonstrated that (**b**) cell islands within the cell infiltrate of *Mmp20*^+/+^Tg incisors also express amelogenin. (**c**) WT, but not (**d**) *Mmp20*^−/−^ secretory stage ameloblasts express MMP20. Interestingly, in *Mmp20*^+/+^Tg incisors, the MMP20 expression pattern (**e**,**f**) was similar to the amelogenin expression pattern (**b**) indicating that the cell infiltrate was composed of cells that may formerly have been secretory stage ameloblasts or their derivatives. Immunofluorescence was performed with antibodies specific for β-catenin and DAPI staining (color was changed from blue to magenta) identified the nuclei in WT and *Mmp20*^+/+^Tg incisors. (**g**–**j**) β-catenin located along the cell membrane in WT ameloblasts, while (**k**–**n**) β-catenin predominantly located to nuclei within *Mmp20*^+/+^Tg ameloblasts. These data suggest that in *Mmp20*^+/+^Tg incisors, cadherin cleavage by MMP20 releases β-catenin from its predominant position adjacent to the plasma membrane and that β-catenin relocates to cell nuclei to promote cell invasion into the enamel space. Ameloblasts, Am; stratum intermedium, SI; enamel space, En; dentin, De; odontoblasts, Od.

We established that bone-related proteins were expressed at higher than normal levels in the *Mmp20*^+/+^Tg incisors but, although no discernable columnar cells were present in the cell infiltrate, we asked if any of these cells were once secretory stage ameloblasts. To answer this question we used fluorescent labeling of frozen sections with antisera specific for amelogenin or MMP20. Although the WT ameloblasts separated from the enamel during sectioning, their apical ends stained strongly for the presence of amelogenin (Fig. 7a). Strikingly, incisors from *Mmp20*^+/+^Tg mice had cell islands present within the infiltrated cells that stained positively for amelogenin (Fig. 7b). To confirm that former secretory stage ameloblasts were present within the cell infiltrate and to determine if any portion of the migrating cells secreted MMP20, we also immunostained to identify cells expressing MMP20. As for amelogenin, ameloblasts from WT incisors stained positively at their apical ends. However, in contrast to amelogenin, a line of MMP20 staining was also observed at the WT dentin-enamel junction (Fig. 7c). Odontoblasts express MMP20, so this was the expected staining pattern. No staining was observed in *Mmp20*^−/−^ mice (Fig. 7d). Like amelogenin, incisors from *Mmp20*^+/+^Tg mice had numerous cell islands within the infiltrate that stained positively for MMP20 (Fig. 7e,f). These results suggest that ameloblasts that are no longer an organized cell layer and that have lost their columnar shape (former ameloblasts), are part of the cell infiltrate present in the enamel space of the *Mmp20* overexpressing mice. These data also suggest that MMP20 assists in the cell migration/invasion process.

### Intracellular β-catenin localization

Cadherins are cell-cell adhesion proteins and we previously demonstrated that cadherins are essential for proper enamel formation^19^ and that MMP20 cleaves both E- and N-cadherins^35^. Since β-catenin is a cell signaling molecule and is part of the complex that binds the intracellular portion of cadherins to the cytoskeleton, we asked if cadherin cleavage by overexpressed MMP20 would release β-catenin from the cadherin-catenin complex.

Fluorescent staining of frozen incisor sections from WT mice revealed that β-catenin predominantly located to the ameloblast cell membrane (Fig. 7g–j). However this did not occur in the *Mmp20*^+/+^Tg infiltrate. Strikingly, in the *Mmp20*^+/+^Tg mice, β-catenin predominantly located to the nuclei of the infiltrating cells (Fig. 7k–n). Together these data suggest that excessive cleavage of cadherins by MMP20 overexpression disrupts ameloblast cell-cell contacts and releases β-catenin, which then translocates to the nucleus to promote cell division and cell invasion into the site where an enamel layer would normally be found.

## Discussion

Here we demonstrate that inducible expression of *Mmp20* in ALC cells enhances invasion through Matrigel coated membranes and results in increased nuclear β-catenin levels (Fig. 2). Previously we engineered a transgenic mouse line that has approximately 4.6 kb of proximal amelogenin promoter to drive *Mmp20* expression^21^. Although the most highly expressed transgene almost completely reverted the dysplastic enamel phenotype in the *Mmp20* ablated mice, it unexpectedly resulted in an almost complete absence of a true enamel layer when present in the WT background^21^. To determine what caused this, we examined longitudinal incisor sections from MMP20 overexpressing mice and found a massive cell infiltrate with associated ectopic mineralized material filling the space where enamel normally locates. Additionally, some residual ameloblasts weaved in and out of the plane of section. This enamel space contained ectopic bone-like calcifications and collagen with cells resembling osteocytes embedded within the bone-like mineral (Fig. 4). qPCR analysis demonstrated that bone-related proteins were expressed at significantly higher levels than normal in incisors from the *Mmp20* overexpressing mice (Fig. 6). The cell infiltrate contained fibroblasts, epithelial cells and cells undergoing cell division (Fig. 5) and many of these non-columnar cells expressed amelogenin and/or MMP20 indicating that they were likely from the epithelial-derived enamel organ (Fig. 7).

The ectopic bone-like mineralization was unexpected. However, it was previously demonstrated that Wnt signaling, perhaps through β-catenin interactions with Runx2, plays an important role in osteogenic differentiation^47^. Additionally, we found that fibroblasts were present within the cell infiltrate and it is known that fibroblasts have osteogenic potential^48^,^49^ and can express Runx2^50^. So, it is plausible that Wnt signaling initiated osteogenic differentiation of fibroblasts, which were responsible for the ectopic collagen containing bone-like calcifications present within the enamel space of the MMP20 overexpressing mice.

We performed multiple tissue expression analysis and demonstrated that Tg expression was restricted to developing teeth and was downregulated in a similar manner to WT MMP20 as development progressed through the maturation stage (Supplementary Table S1, Fig. 3). Therefore, unrestricted *Mmp20* expression was not responsible for the cell infiltrate.

We also showed that MMP20 overexpression initiated β-catenin signaling. Since MMP20 cleaves cadherins and releases β-catenin that was associated with these cadherins, we asked if this signaling pathway could be responsible for the observed cell pathology. β-catenin is a component of the complex that binds the intracellular cadherin domain to the cell cytoskeleton^16^. When matrix metalloproteinases (MMPs) cleave an extracellular classic cadherin domain, β-catenin is removed from its position near the cell membrane and may translocate to the cell nucleus^25^,^26^,^27^,^28^,^29^,^30^,^31^. This translocation is associated with cell movement/invasion^51^,^52^. Previously, MMP20 was shown to be expressed by squamous cell carcinomas^53^,^54^ and its expression was associated with metastasis to lymph nodes^53^. These carcinoma results support our data demonstrating increased cell invasion with increased MMP20 expression.

However, we did explore the possibility that the cell invasion occurred by other means. To address whether cell invasion into the incisor enamel space occurred in MMP20 overexpressing mice because of an autoimmune inflammatory response initiated by the release of enamel matrix proteins, we asked if amelogenin antibodies and/or amelogenin protein were present in the serum of the overexpressing mice. ELISA assays performed on mouse serum detected no anti-amelogenin antibodies and immunoblots did not detect amelogenin. These results suggest that an autoimmune response was not likely responsible for the massive cell invasion into the enamel space.

**Figure 8:**
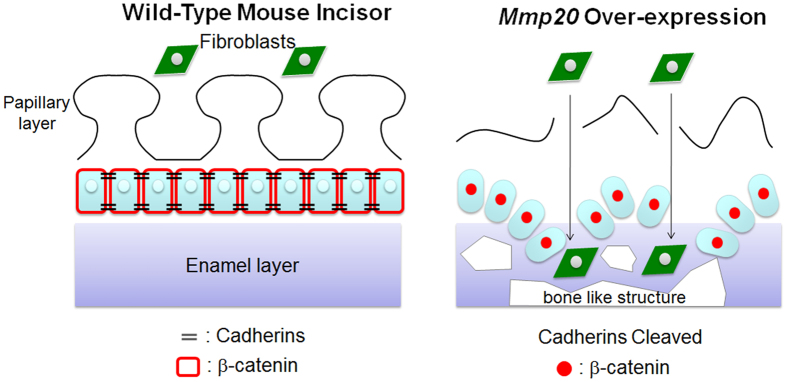
Schematic depicting how fibroblasts and epithelial cells may gain entry into the *Mmp20*^+/+^Tg incisor enamel space. *Mmp20* overexpression results in increased cadherin cleavage, which may disrupt the ameloblast layer. When the ameloblast layer is disrupted, fibroblasts near the capillary basement membranes within the papillary layer may become free to move into the enamel space. These invading fibroblasts may contribute to the formation of the ectopic bone-like formations found within the *Mmp20*^+/+^Tg incisor enamel space. Epithelial and fibroblast cell migration into the enamel space may be enhanced by β-catenin translocation to cell nuclei due to extensive cadherin cleavage and resultant β-catenin release by the MMP20 overexpressing mice.

Interestingly, cohorts of ameloblasts naturally migrate during the secretory stage of enamel development. The enamel rod is the mineralized trail of the ameloblast that formed it and accumulating evidence suggests that MMP20 cleaves cadherins so that groups of ameloblasts can slide by one another to establishing the rodent decussating enamel rod pattern or the entwined gnarled prism pattern seen in human molars^16^. In *Mmp20* ablated mice the rod pattern is either nonexistent or is severely dysplastic. Previously we showed that a highly expressing *Mmp20* transgene significantly ameliorated the enamel rod pattern and hardness in *Mmp20* ablated mice^21^. This suggests that MMP20 plays a significant role in ameloblast movement, which is necessary to form enamel rod patterns. Here, we demonstrated that in incisors from WT mice, β-catenin predominantly located adjacent to the ameloblast cell membrane. In contrast, but consistent with the cell infiltrate invasion into the enamel space, we found that β-catenin predominantly located to the nuclei of the invading cells in incisors from *Mmp20*^+/+^Tg mice. A schematic synopsis is presented demonstrating how MMP20 overexpression may lead to fibroblast and epithelial cell invasion into the enamel space (Fig. 8).

Taken together, our data suggest that in addition to its well characterized role in cleaving enamel matrix proteins, MMP20 also plays an essential role in ameloblast movement. When expressed at normal levels, MMP20 cleaves cadherin cell-cell junctions and releases a limited amount of β-catenin necessary to regulate movement of ameloblasts as the enamel rod pattern is formed. If *Mmp20* is ablated, the rod pattern is absent or severely malformed. In contrast, if MMP20 is overexpressed, cadherin cleavage and β-catenin release is dramatically enhanced resulting in a large increase in migration/invasion that causes a mass of cells to invade the enamel space. Since fibroblasts are present within the cell mass, it appears that the ameloblast layer becomes discontinuous and allows these fibroblasts in the peripheral connective tissue regions to move freely. We conclude that MMP20 is essential for enamel rod pattern formation through a signaling mechanism that includes cleavage of cadherins and subsequent translocation of β-catenin to ameloblast nuclei necessary to promote cell migration.

## Methods

### Animals

All animals were housed in Association for Assessment and Accreditation of Laboratory Animal Care (AAALAC) accredited facilities (animal welfare assurance number: A3051-01) and were treated humanely based on a protocol approved by The Forsyth Institute Institutional Animal Care and Use Committee (IACUC). Experimental protocols were designed along institutional and National Institutes of Health guidelines for the humane use of animals. Previously we identified three different transgenic mice (Tg) that express MMP20 at low, intermediate and high levels^21^. All mice in these experiments had the transgene (Tg24) that expressed high levels of MMP20 in incisors.

### Inducible Mmp20 Expression Construct, Transfections and Culture Conditions

A hemagglutinin epitope tag (HA) comprising the amino acid sequence YDVPDYA and the nucleotide sequence TACGACGTACCAGACTACGCA was added to the 3′ end of the mouse *Mmp20* cDNA immediately before the stop codon. The cDNA also encoded the autoactivating mutant (Val^101^-Gly^101^) so that MMP20 was secreted as an active proteinase^28^,^55^,^56^. The *Tet*-Off advanced inducible gene expression system (Clontech, Mountain View, CA) was used for transgenic MMP20 expression. Mouse *Mmp20* cDNA was ligated into KpnI-NotI sites of the pTRE-Tight vector (hygromycin resistant). A p*Tet*-Off advanced vector expressing the tetracycline-controlled transcriptional transactivator (neomycin resistant) was transfected (Xfect transfection reagent, Clontech) into the mouse ameloblast-lineage cell (ALC) line^22^. Hygromycin-resistant colonies were selected and cloned. *Mmp20* expression levels for each resulting cell line were assessed by qPCR, immunoblots and casein zymography.

### Isolation of Nuclear and Cytoplastic Cell Extracts

After removing the media, cells were rinsed with cold PBS twice, scraped from the plates, and pelleted by centrifugation at 1000 rpm for 5 min. cell pellets were suspended in buffer A [10 mM HEPES, pH 7.9, 10 mMKCl, 0.1 mM EDTA] with Halt Protease and Phosphatase Inhibitor Single-Use Cocktail (Thermo Scientific). After 10 min incubation on ice, 0.1% NP-40 was added and incubated for 5 min at room temperature followed by vigorous vortexing for 20 sec. The cells were spun down at 2000 rpm for 1.5 min at 4 °C. The supernatant contained the cytoplasmic fraction. The nuclear pellets were washed with buffer A and spun down for 1.5 min at 3000 rpm at 4 °C. The pellets were resuspended in buffer B [20 mM HEPES, pH 7.9, 0.4 M NaCl, 1 mM EDTA] containing Halt Protease and Phosphatase Inhibitor Single-Use Cocktail and were vigorously vortexed for 30 min at 4 °C and spun down at 14000 rpm for 10 min at 4 °C. After these steps, the nuclear fraction was present in the supernatant. Nuclear and cytoplasmic fractions were extracted for immunoblot analysis.

### Immunoblots and Zymography

Stably transfected ALC cells were cultured in serum free medium with or without 100 ng/ml Dox plus Halt Protease and Phosphatase Inhibitor Single-Use Cocktail to inhibit non-MMP proteolytic activity that may degrade expressed MMP20. Immunoblots were accomplished as described previously^4^,^21^. Briefly, culture medium proteins were concentrated by filtration of media through Amicon Ultra-0.5 mL 10 K (Millipore) followed by suspension of the remaining proteins in sample buffer. Proteins were also extracted from first molar enamel organs from 5-day-old mice for quantification of E- and N-cadherins. These proteins were eluted into sample buffer and equal amounts of protein (25 mg of molar proteins) were loaded into each lane and run on SDS-PAGE for transfer to a nitrocellulose membrane. Immunoblots were performed with antibody specific for β-catenin, α-tubulin, histone H3, E-cadherin (Cell Signaling Technology, Danvers, MA), HA-tag or N-cadherin (ThermoFisher Scientific, Waltham, MA). Casein zymography was performed as previously described^4^,^7^,^21^,^57^. Briefly, after electrophoresis, gels were washed twice for 30 min in 2.5% Triton X-100. Gels were incubated for 2 days at 37 °C in 50 mM Tris-HCl buffer (pH 7.2) containing 10 mM CaCl_2_ and were then stained with Coomassie Brilliant Blue R-250 solution for 15 min and destained with 30% methanol and 10% acetic acid until clear bands of substrate lysis were observed.

### Cell Proliferation Assays

Proliferation of *Tet*-Off-*Mmp20* cells was measured using the cell proliferation reagent WST-1 (Roche). Five thousand cells were seeded per well into 96-well plates and incubated at 37 °C with or without 100 ng/ml Dox in a CO_2_ incubator for up to 72 h. WST-1 was next added to the culture medium and incubated for 2 h at 37 °C. The amount of reduced tetrazolium was determined by measuring absorbance at 450 nm in a microplate reader (Polar Star Optima, BMG Labtech, Cary, NC). These experiments were performed in triplicate and were repeated twice.

### Transwell Invasion Assay

*Tet*-Off-*Mmp20* cells were added onto filter well inserts with a top layer of Matrigel (8 μm pore size; Corning, Corning, NY) and placed into each culture well. Cells were cultured with protease inhibitors that excluded MMP inhibitors and were treated with or without 100 ng/ml Dox for the indicated time periods in a cell culture incubator at 37 °C with 5% CO_2_. Cells on the top of the filter were wiped off, and the filter was fixed in formalin and stained with hematoxylin. The total number of cells that migrated to the opposite side of the filter was determined by averaging the number of cells counted in five random microscope fields (magnification 200X). These experiments were performed in triplicate and were repeated three times.

### Immunocytofluroescence

*Tet*-Off-*Mmp20* cells were seeded on glass coverslips. The cells were incubated with or without 100 ng/ml Dox for 3 days in a cell culture incubator at 37 °C with 5% CO_2_ and were fixed in 10% formalin. After two washes with PBS, the cells were permeabilized with 0.1% TritonX-100 in PBS and blocked by incubation with 5% pre-immune goat serum in PBS for 30 min followed by overnight incubation with antibodies specific for β-catenin (1:100, Cell Signaling). The cells were washed three times and incubated for 1 hour with Alexa Fluor 488 goat anti-rabbit IgG antibody (1:200) in 5% pre-immune goat serum in PBS. The cells were then washed twice with PBS, incubated with 4′,6-diamidino-2-phenylindole (DAPI, NucBlue Fixed Cell Stain, Life Technologies, Grand Island, NY) for 5 min, and washed twice with PBS and once with water before mounting with ProLong Gold (Life Technologies). Sections were examined with a fluorescent microscope.

### Histology

Seven-week-old mice were euthanized and their skinned heads were fixed in 10% zinc formalin overnight at room temperature, washed with PBS and decalcified in 20% sodium citrate, 10% formic acid solution for 2 weeks at room temperature. The tissues were dehydrated in a graded series of ethanol washes and embedded in paraffin for sectioning. Selected deparaffinized and rehydrated sections were stained with haematoxylin/eosin. Immunohistochemistry was performed as previously described^19^. Dewaxed and rehydrated sections from mouse jaws were subjected to microwave activated antigen retrieval in 10 mM sodium citrate pH 6.0. Endogenous peroxidase was quenched with 3% hydrogen peroxide in methanol. Sections were incubated in blocking agent for 30 min followed by overnight incubation with antibodies specific for keratin-14 (1:500, Thermo Scientific), Ki67 (1:100, Thermo Scientific), or FSP1 (1:800, Cell Signaling). Staining was visualized by incubation with the Vecta Stain ABC Kit (Vector Laboratories, Burlingame, CA) peroxidase-conjugated antibody and Sigma Fast 3,3′-diaminobenzidine substrate (Sigma, St. Louis, MO). Sections were counterstained with haematoxylin and examined by light microscopy.

For frozen sections, mouse heads were fixed with 4% paraformaldehyde overnight at 4 °C. Tissues were decalcified with 250 mM EDTA/PBS for 2 weeks. After decalcification the tissues were immersed in 30% sucrose overnight and embedded into OCT compound. Eight μm-thick sections were cut on a cryostat (Shandon Cryotome FSE, Thermo Scientific) and incubated in blocking agent for 30 min followed by overnight incubation at 4 °C with antibodies specific for amelogenin (1:500)^58^, MMP20 (1:200, Abcam) or β-catenin (1:100, Cell Signaling). Sections were examined by confocal microscopy (Zeiss LSM 780).

### Enzyme-Linked Immunosorbent Assays (ELISA)

Serum was collected from wild-type (WT) or *Mmp20*^+/+^Tg mice^21^ and recombinant mouse amelogenin were bound to 96-well ELISA plates. Mouse serum (1/100 dilution) was applied to the plates and incubated for 2 h. After washing, horseradish peroxidase-conjugated goat anti-mouse IgG (Sigma) (1/2000 dilution) was added to the plates and incubated for 20 min. Reactions were terminated by the addition of 2N H_2_SO_4_ and absorbance at 485 nm was determined with a microplate reader (Polar Star Optima).

### Quantitative Real-Time PCR (qPCR)

RNA extracted from adult mouse incisor enamel organs or from first molar enamel organs from 7, 9 or 10 day-old pups were used to determine relative expression levels of *Mmp20, Amelx* (amelogenin)*, Dspp* (dentin sialophosphoprotein)*, Col1a1* (collagen type I α-chain)*, Ibsp* (bone sailoprotein)*, Bglap* (osteocalcin), *Alpl* (alkaline phosphatase), *Sox9*, and *Col2a1* (collagen type II α-chain) as a function of the stably expressed internal reference control gene *Rn18*s (18S rRNA) as previously described^59^,^60^. Primers that amplify each gene’s cDNA are listed in Supplementary Table S2. Reactions were performed on a Roche LightCycler 480 programmed as follows: 3 min at 95 °C for initial denaturation, and 95 °C 15 sec, 58 °C 15 sec, 72 °C 15 sec for 40 cycles, followed by a melt curve. Each data point was obtained by triplicate qPCR analysis. Data are presented as the mean ± SD for three independent experiments.

### Statistics

T-tests were performed for analyzing the significance of qPCR and cell invasion assay results.

## Additional Information

**How to cite this article**: Shin, M. *et al*. Murine matrix metalloproteinase-20 overexpression stimulates cell invasion into the enamel layer via enhanced Wnt signaling. *Sci. Rep.* **6**, 29492; doi: 10.1038/srep29492 (2016).

## Supplementary Material

Supplementary Information
