# Supplementary material for: Murine matrix metalloproteinase-20 overexpression stimulates cell invasion into the enamel layer via enhanced Wnt signaling
Source: Sci Rep. 2016 Jul 11;6:29492. doi: 10.1038/srep29492 (PMC4941722; doi:10.1038/srep29492)
Supplement: Supplementary Information [file srep29492-s1.doc]

SREP-16-08624

Title: Murine matrix metalloproteinase-20 overexpression stimulates cell invasion into the enamel layer via enhanced Wnt signaling.

By: Masashi Shin, Maiko Suzuki, Xiaomu Guan, Charles E. Smith and John D. Bartlett

**Supplementary Table S1. Tissue-Specific qPCR to Assess *Mmp20* Expression.**

*Mmp20* cycle threshold *Rn18s* cycle threshold


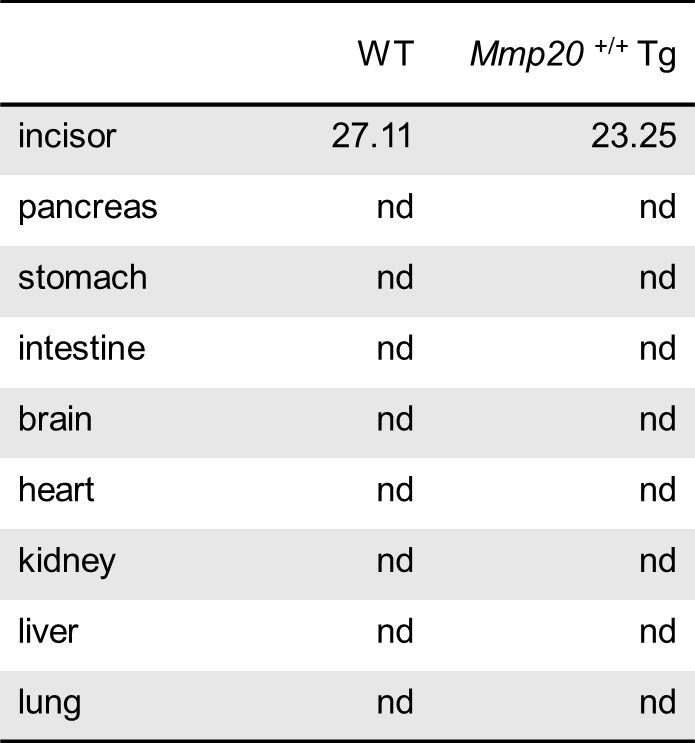

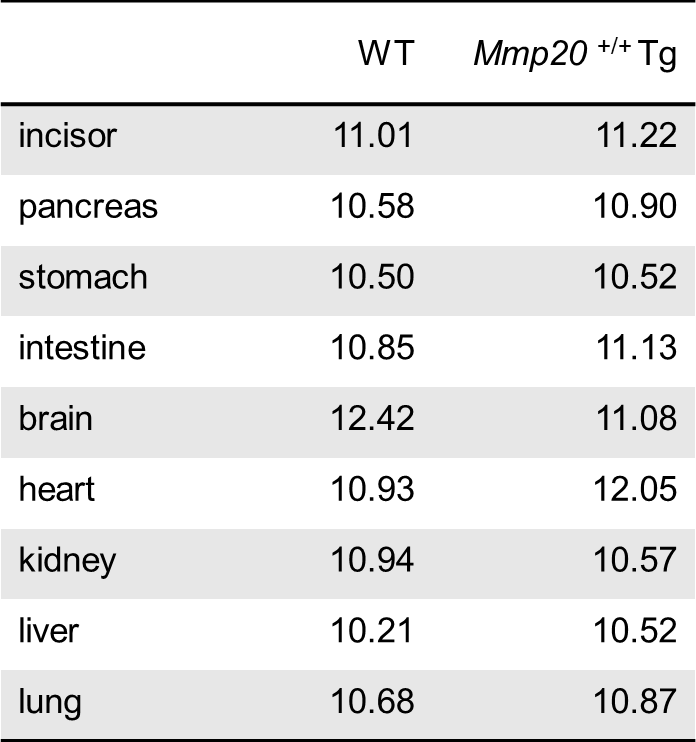


nd, not detected

**Supplementary Table S2. Primers Used for Quantitative Real-Time PCR Results.**


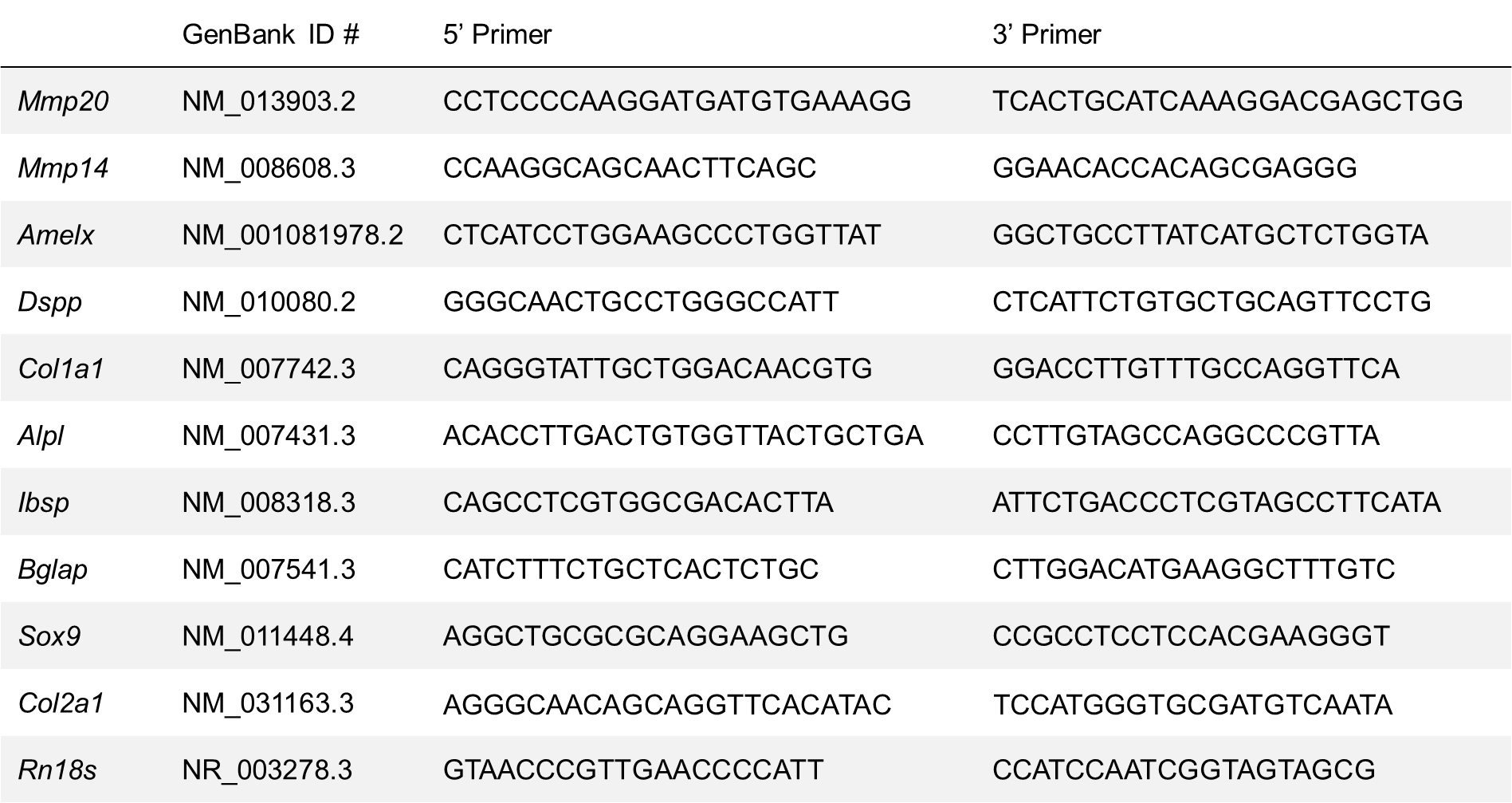


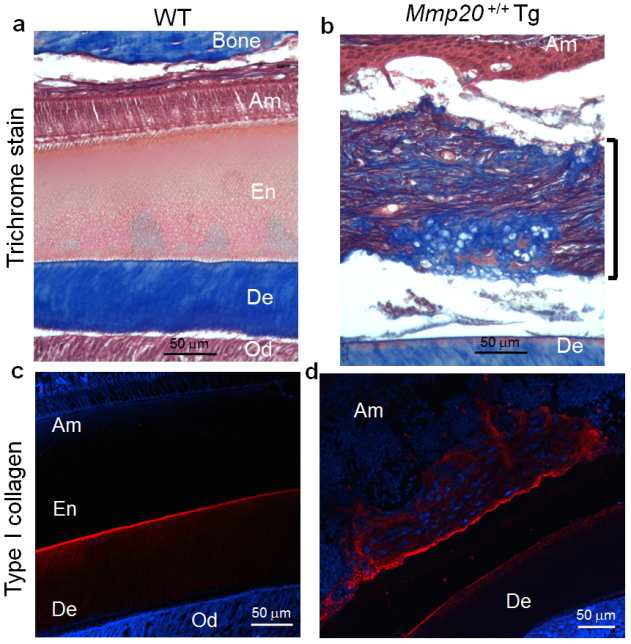


**Supplementary Figure S1.** **Trichrome staining and immunohistochemical analysis of incisor sections from wild-type (WT) and Mmp20^+/+^Tg mice.** Trichrome staining of (**a**) WT and (**b**) *Mmp20^+/+^*Tg developing incisor enamel revealed that poorly organized collagen was present within the cell infiltrates of the Tg mice. (**c, d**) Immunohistochemical staining of Type I collagen was performed on longitudinal incisor sections from WT and *Mmp20^+/+^*Tg mice. (**C**) In WT mice, staining for Type I collagen was observed along the dentin-enamel junction, but staining was not observed within the enamel space. (**d**) In contrast, *Mmp20^+/+^*Tg incisors had positive type I collagen staining within the enamel space presumably near or within the observed ectopic calcifications. Ameloblasts, Am; enamel space, En; dentin, De; odontoblasts, Od.


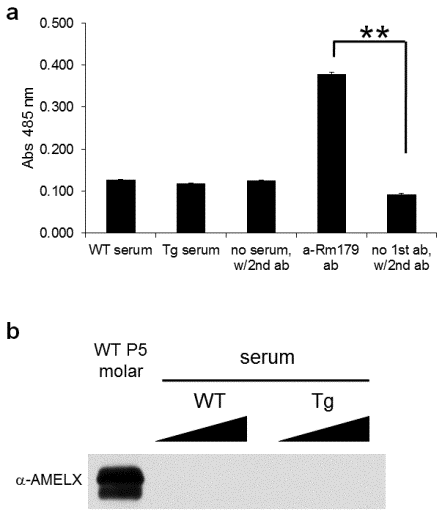


**Supplementary Figure S2. The enamel organ pathology observed in the *Mmp20^+/+^Tg* mice (Tg) does not appear to result from an amelogenin induced humoral immune response.** (**a**) ELISA assays were performed to identify antibodies specific for recombinant mouse amelogenin composed of 179 amino acids (rM179). Wild-type (WT) serum served as the negative control. The assay without serum but with the secondary antibody included, identified the background absorbance level. The positive control showed that when exogenous anti-amelogenin antisera was included, this resulted in a highly significant increase in absorbance. Conversely, when the anti-amelogenin antiserea was omitted, the absorbance dropped to background levels. Note that in serum from Tg mice, no antibodies specific for amelogenin were present. The presented data were the mean ± SD performed in triplicate with three experimental replicates, **P<0.01. (**b**) To confirm that amelogenin did not leak into the blood stream of Tg mice, 0.1 and 0.2 μl of WT and Tg mouse serum were immunoblotted with anti-amelogenin antibody. Protein extracted from the molars of five-day old (P5) mice served as a positive control. Amelogenin was not present in serum from Tg mice.


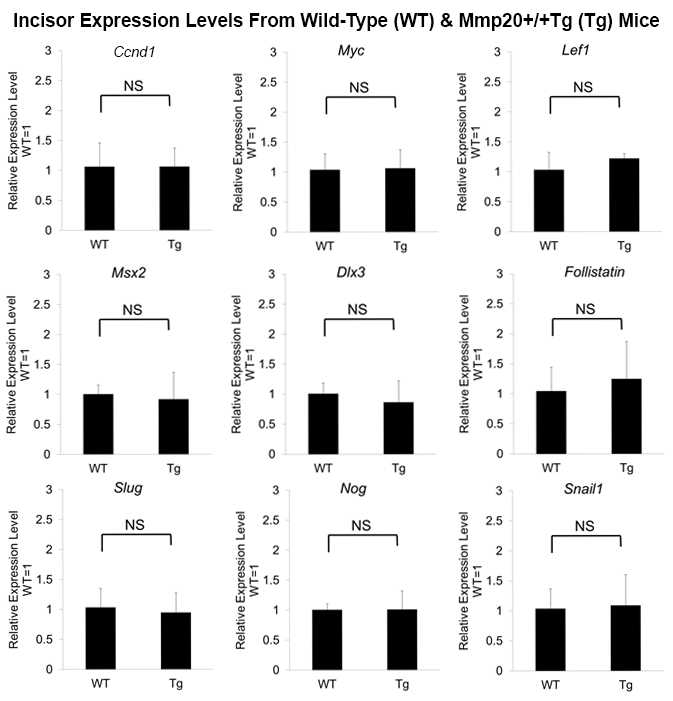


**Supplementary Figure S3. *Wnt* responsive genes were not significantly induced in incisors from *Mmp20^+/+^Tg* mice when compared to incisors from wild-type mice.** This surprising result may have occurred because only the ameloblasts of the enamel organ secrete MMP20 and the entire enamel organ was assessed for gene expression. Therefore, subtle differences may not be detectible. *Gapdh* was the reference control gene. NS, not significant.
